# Experiences of change following a blended intervention for adults with ADHD and emotion dysregulation: a qualitative interview study

**DOI:** 10.1186/s12888-025-06476-1

**Published:** 2025-01-20

**Authors:** Emilie S. Nordby, Viktor Schønning, Alice Barnes, Hayley Denyer, Jonna Kuntsi, Astri J. Lundervold, Frode Guribye

**Affiliations:** 1 Division of Psychiatry, Haukeland University Hospital, Bergen, Norway; 2 Department of Biological and Medical Psychology, University of Bergen, Bergen, Norway; 3 Social, Genetic and Developmental Psychiatry Centre, Institute of Psychiatry, Psychology and Neuroscience, King’s College London, London, UK; 4 Department of Information Science and Media Studies, University of Bergen, Bergen, Norway

**Keywords:** ADHD, Emotion regulation, Psychological interventions, Blended interventions, Digital interventions, Qualitative research

## Abstract

**Background:**

Emotion dysregulation commonly co-occurs with attention-deficit hyperactivity disorder (ADHD), leading to a range of negative outcomes. While psychological interventions have shown promise in bringing about positive changes in emotional and cognitive domains, there is still limited knowledge on the subjective experiences of change among the participants in these interventions.

**Aim:**

The present study explores the experiences of adults with ADHD who had participated in a blended digital and face-to-face intervention aimed at improving emotion dysregulation. The study focuses on understanding their experiences of change and identifying contributors to change.

**Methods:**

A total of 9 adults with ADHD participated in individual semi-structured, in-depth interviews following their participation in the intervention. The interviews were analyzed using thematic analysis.

**Results:**

The thematic analysis resulted in three core themes. The first theme, ‘*perceiving change’*, represents changes that the participants experienced, with four subthemes: 1a) *being in control*,* 1b) feeling aware*, 1c) *accepting oneself and one’s emotions* and 1d) *gaining insight and knowledge*. The second theme, ‘*supporting change*’, captures factors that supported the participants’ changes, with five subthemes: 2a) *acquiring skills*,* 2b) being in it together*,* 2c) therapist guidance*,* 2d) finding motivation 2e) putting it into practice*. Lastly, the third theme, ‘*sustaining change*’, includes aspects important to maintain change, with two subthemes: 3a) *working consistently* and *3b) giving it time*.

**Conclusions:**

The findings show that the participants experienced various changes related to the management of their emotions following their participation in the intervention. Change was perceived as a multifaceted process, supported by internal factors such as motivation and engagement, along with external factors such as support from the other group members and the therapists. Taken together, the findings from the study could be important to the development of psychological interventions for adults with ADHD and may provide valuable knowledge to clinicians and policymakers.

**Trial registration:**

Registered on 21st November 2022 at ClinicalTrials.gov. ClinicalTrials.gov ID: NCT05644028.

## Background

Attention-deficit hyperactivity disorder (ADHD) is a heterogeneous neurodevelopmental condition with a high persistence rate into adulthood [1, 2]. The primary diagnostic criteria include symptoms of inattention, hyperactivity, and impulsivity, but a large proportion of individuals with ADHD also experience challenges related to emotion dysregulation [3]. For instance, some studies indicate that adults with ADHD tend to use more maladaptive emotion regulation strategies, such as suppression and avoidance, and fewer adaptive emotion regulation strategies, such as cognitive reappraisal [4–6]. In addition, their emotional experiences are often more intense than those of their peers, with rapidly shifting emotional states, which may also make them more easily emotionally overwhelmed [7]. Emotion dysregulation seems to be more prevalent in adults than in children with ADHD, with between 34 and 70% of adults and 24–50% of children experiencing such challenges [3]. Furthermore, individuals with ADHD and co-occurring symptoms of emotion dysregulation tend to experience more severe impairment than individuals without this co-occurrence [3, 8]. Co-occurring symptoms of emotion dysregulation are associated with more persistent ADHD symptoms, more severe social problems, a higher frequency of psychiatric comorbidity, and greater risk of more severe outcomes such as self-harm and suicidal ideation [9–12]. As such, it is important to provide interventions that address these challenges in ADHD.

Adults with ADHD have few treatment options for co-occurring symptoms of emotion dysregulation. Traditional pharmacological interventions for ADHD are known to reduce the severity of core symptoms of inattention and hyperactivity-impulsivity, but tend to be less effective at remediating symptoms of emotion dysregulation [13]. Clinical guidelines emphasize that adults with ADHD who do not experience sufficient clinical improvement following pharmacological treatment should be offered psychological interventions as part of their treatment course [14].

As emotion dysregulation is a common challenge across many mental health conditions, there are several psychological approaches aimed at addressing this difficulty. In particular, dialectical behavior therapy (DBT) has been shown to be an effective approach for emotion dysregulation [15]. DBT is assumed to improve emotion regulation by introducing skills that help clients to downregulate their emotional reactions, endure emotions, and have more adaptive behavioral responses in challenging situations [16]. DBT has traditionally been applied to people with borderline personality disorder, but components of DBT have been adapted to other mental health conditions, including ADHD [17–23]. Studies of DBT-inspired interventions for ADHD have shown improvements in emotion dysregulation from pre- to post-treatment [18, 20, 22]. However, when compared to treatment-as-usual (TAU), no significant differences in emotion dysregulation were identified between DBT and TAU among adults with ADHD [17]. There have also been studies reporting improvements in emotion regulation among adults with ADHD from pre- to post-treatment in studies of other psychological intervention frameworks, such as mindfulness-based approaches and goal management training [24, 25].

Recently, there has been an increase in the use of digital interventions for various mental health conditions, including ADHD [26]. There are several advantages to a digital treatment format, such as making psychological interventions more accessible, flexible, and cost-effective [27]. A meta-analysis on digital interventions for children and adults with ADHD concluded that digital interventions were effective in reducing inattention and improving social functioning as compared to waitlist controls [26]. Furthermore, qualitative research suggests that adults with ADHD perceive some benefits of including technology in the management of ADHD, for instance by offering more insight into their own health-related data [28]. At the same time, there can also be challenges related to digital interventions, such as non-adherence, a more superficial therapeutic bond, or missing non-verbal cues [29–31]. An approach to utilize the benefits and minimize the downsides of digital interventions is to combine them with face-to-face interventions in a blended approach. A blended approach has the potential to save time and resources, as well as to maintain intervention effects long-term [32].

While the current body of literature, primarily grounded in quantitative research, documents the clinical effects of psychological interventions for adults with ADHD, less is known about the participants’ experiences of such therapeutic effects, especially in digital interventions and interventions that target emotion dysregulation. To explore this, a qualitative design might be particularly valuable, as it gives in-depth knowledge of the lived experience of the people taking part in these interventions [33]. Further, given that therapeutic change is inherently experienced in a subjective manner, exploring change from the first-person perspective can provide insights that cannot be captured using quantitative methods. In addition, when it comes to interventions designed for neurodivergent individuals, it is crucial that the perspectives of the participants themselves are included in both research and clinical practice [34].

Few studies have explored the experiences of adults with ADHD engaged in psychological interventions. However, some recent studies employing a qualitative design have given insight into the experiences of change among participants of such interventions. For instance, Janssen et al. [35] conducted focus groups and individual interviews to examine the experiences of adults with ADHD taking part in a mindfulness-based intervention. Their study identified the following eight stages in the process of change: *stopping*,* noticing*,* allowing*,* insight*,* changing perspective*,* self-regulation*,* changing behavior*, and *experiencing effects*. In addition, some specific facilitators in the intervention were identified, including the support from a partner, contact with others with ADHD, and diversity in exercises. There were also some barriers, such as life stressors, lack of time, lack of repetition, too much homework, short course length, and ADHD symptoms [35]. Similarly, Seery et al. [36] explored the experiences of adults with ADHD taking part in an internet-based intervention combining psychoeducation with acceptance and commitment therapy (ACT). This study found that participants attributed behavioral change to psychoeducation on ACT processes [36]. Additionally, Sehlin et al. [37] explored the experiences of young adults with ADHD and autism spectrum disorder who participated in an 8-week internet-delivered intervention. In this study, the participants expressed that chatting with a clinician contributed to positive change, for example by calming their emotions [37]. Taken together, these studies provide valuable insight into the experiences of change among people with ADHD taking part in psychological interventions. Still, to our knowledge, there are no studies that have examined experiences of adults with ADHD taking part in a psychological intervention specifically designed to address emotion dysregulation.

The present study aimed to investigate the first-person perspective of change following a blended digital and face-to-face intervention targeting emotion dysregulation in adults with ADHD. By using a qualitative methodology, the study not only seeks to explore the experienced changes, but also the contributors to these changes from the perspective of the participants themselves. This knowledge may be useful in guiding the development of psychological interventions for ADHD, particularly those integrating technology and/or targeting emotion dysregulation among adults with ADHD.

## Methods

### Study context

The study was conducted in Bergen, the second largest city in Norway. In recent years, there has been an increasing number of individuals diagnosed with ADHD in Norway [38]. Within Norway, adults with ADHD are also increasingly more often seeking non-pharmacological treatment options [39]. However, to date, there are few psychological options available for adults with ADHD within the public mental healthcare system in Norway [39].

All participants in this study had recently participated in a feasibility study of the “Emotion Regulation Intervention for ADHD” (ERIA), which is an 8-week blended intervention designed to improve emotion regulation skills among adults with ADHD [40]. The intervention builds on principles from DBT skills training and positive psychology. It combines weekly face-to-face group sessions and a digital companion app for skills training in-between sessions. The groups were closed, including 6–8 adults with ADHD, and led by two clinical psychologists.

The participants were recruited through the Norwegian ADHD patient association, which distributed information about the study through email and their social media channels.

### Participants

All participants from the feasibility study were invited to take part in qualitative interviews. Inclusion criteria for the feasibility study were: (1) age above 18 years, (2) a diagnosis of ADHD, (3) difficulties with emotion dysregulation, as indicated by a score above 80 on the difficulties in emotion dysregulation scale (DERS), (4) access to a computer or smartphone, and (5) ability to attend weekly group sessions in Bergen, Norway. The exclusion criteria were any of the following: 1) a high risk of suicidality, as indicated by (a) suicide attempt within the last year, (b) previous suicide attempt and current suicidal ideations, or (c) current suicidal ideations and preferred method and plan; 2) presence of other severe mental disorders, including substance abuse, psychosis, and major depressive disorder as assessed by a clinical psychologist with the MINI international neuropsychiatric interview [41]; and 3) concurrent participation in a psychological treatment intervention.

### Interviews

The interviews were conducted between the 29th of March and the 17th of April 2023, all within 6 weeks after the participants had completed the intervention. The interviews took place at the psychiatric outpatient clinic at the University of Bergen. The interviews had durations between 17 min and 36 min, with a mean interview length of 27 min.

In the interviews, the participants were asked whether they had experienced any changes following the intervention, whether they experienced positive or negative changes, and what these changes included. They were further asked about what they believed contributed to these changes, and whether changes were related to the program or other factors. In addition, the participants were asked to evaluate what aspects of the intervention they found useful, and what aspects of the intervention they found problematic or challenging. Lastly, they were asked about missing elements in the intervention and recommendations for improvements.

The interviews were conducted by a research assistant who was also a clinical psychology student. The interviewer had not met the participants previously nor been involved in the development of the intervention. This approach was chosen to reduce bias and encourage participants to share their true experiences with the intervention. The interviews were audio-recorded and transcribed verbatim by the interviewer. Transcriptions were then checked for accuracy by the first author by comparing sections of the transcriptions to the audio-recording. The audio-recordings were deleted once the transcription was completed and all identifiable information was removed from the transcripts.

### Data analysis

The interviews were analyzed using a thematic analysis within a hermeneutic phenomenological framework. Hermeneutic phenomenology is a commonly used theoretical framework within health research that combines the descriptive focus of phenomenology with the interpretive lens of hermeneutics to better understand the individual experience [42, 43]. Thematic analysis is a flexible method within qualitative research that seeks to identify and analyze patterns in the data material [44]. The thematic analysis in this study followed the six steps described by Braun and Clarke, which include: (1) becoming familiar with the data; (2) generation of codes, (3) searching for themes, (4) reviewing themes, (5) finalizing themes and (6) writing a report [44].

The initial coding was conducted by the first and second author. Prior to the data analysis, both authors wrote notes on their individual preconceptions and presumptions to uphold awareness and reflexivity regarding their own biases. When conducting the coding, they strove to keep the codes close to the empirical material, and in this way keeping the initial codes close to the participants’ accounts of their experiences. The material was coded with the research questions: “what changes did the participants experience following the intervention” and “what contributed to these changes?”. All interviews were coded twice, separately by the first and the second author, and a subset of the interviews were coded a third time by the last author. Subsequently, the authors’ codes were compared to ensure coherence and consistency in the codes. An overview of all the codes was then created and placed within preliminary categories. Following the coding, the next step was to search for themes. When looking for themes, we searched for broader patterns in the data and examined how the codes were related to one another, and if there were any overarching themes representing subsets of the codes. In creating the themes, the first and last author had an analytic meeting to discuss codes, interpretation, and overarching themes. Following this meeting, an initial thematic structure was created. The thematic structure was then reviewed and discussed in a series of meetings. The final thematic structure consisted of 3 core themes and 11 subthemes. To exemplify a theme or a subtheme, participant quotes that were deemed to capture its essence, were extracted from the interview transcriptions, and included in the results section.

### Ethical considerations

The Regional Ethics Committee of Norway, Region West gave its approval for the current study (ID: 494659). The participants gave their informed consent before participating in the study. The participants were informed that they had the right to withdraw at any time during the interview, without providing any reason for their withdrawal. Participating in the interviews was optional and the participants were informed that their participation in the interviews would not affect their participation in the feasibility trial. The participants received 1000 NOK (90 USD) for participating in the feasibility trial as compensation for their time and expenses related to travelling to the clinic. No extra compensation was given for participation in interviews.

### Reflexivity

The authors have engaged in an ongoing examination of their own biases in a reflexive process throughout this study. Both the first and second authors, who conducted the coding, are clinical psychologists, which may have influenced their interpretations, biasing them towards emphasizing the positive effects of psychological interventions. In an attempt to reduce this bias, the last author, coming from a different background, but with expertise in qualitative research, reviewed the codes and coded a subset of the interviews independently, allowing for different perspectives to be integrated in the analytic work. Moreover, the development of the current intervention was a part of the first author’s doctoral work, where the last two authors were supervisors, which makes them more invested in the intervention. As such, this study engaged authors who were not directly involved in the developmental process to ensure that different viewpoints were included. By incorporating these additional perspectives, we hope to enhance the robustness and credibility of the findings.

## Results

Of the 16 participants in the feasibility study, 10 participants completed the post-assessment, and all 10 agreed to take part in the interview following their participation in the program. However, one participant called in sick and was not able to reschedule, leaving 9 participants for the interviews.

See Table 1 for participant characteristics. The sample included 3 men and 6 women with a mean age of 40.8 years, ranging from 28 to 65 years. All participants were intervention completers.

**Table 1:** Participants characteristics (*n* = 9)

Characteristic	Sample
Education, n (%)	
* High School*	4 (44%)
* Higher education (University/College)*	5 (56%)
Employment, n (%)	
* Full-time employed or student*	4 (44%)
* Work allowance or sick leave*	4 (44%)
* Disability pension*	1 (11%)
Emotion dysregulation (DERS), *M (SD)*	123 (18.1)

Note. *n* = number of participants, DERS = Difficulties in Emotion Regulation Scale, M = mean, SD = standard deviation

**Fig. 1:**
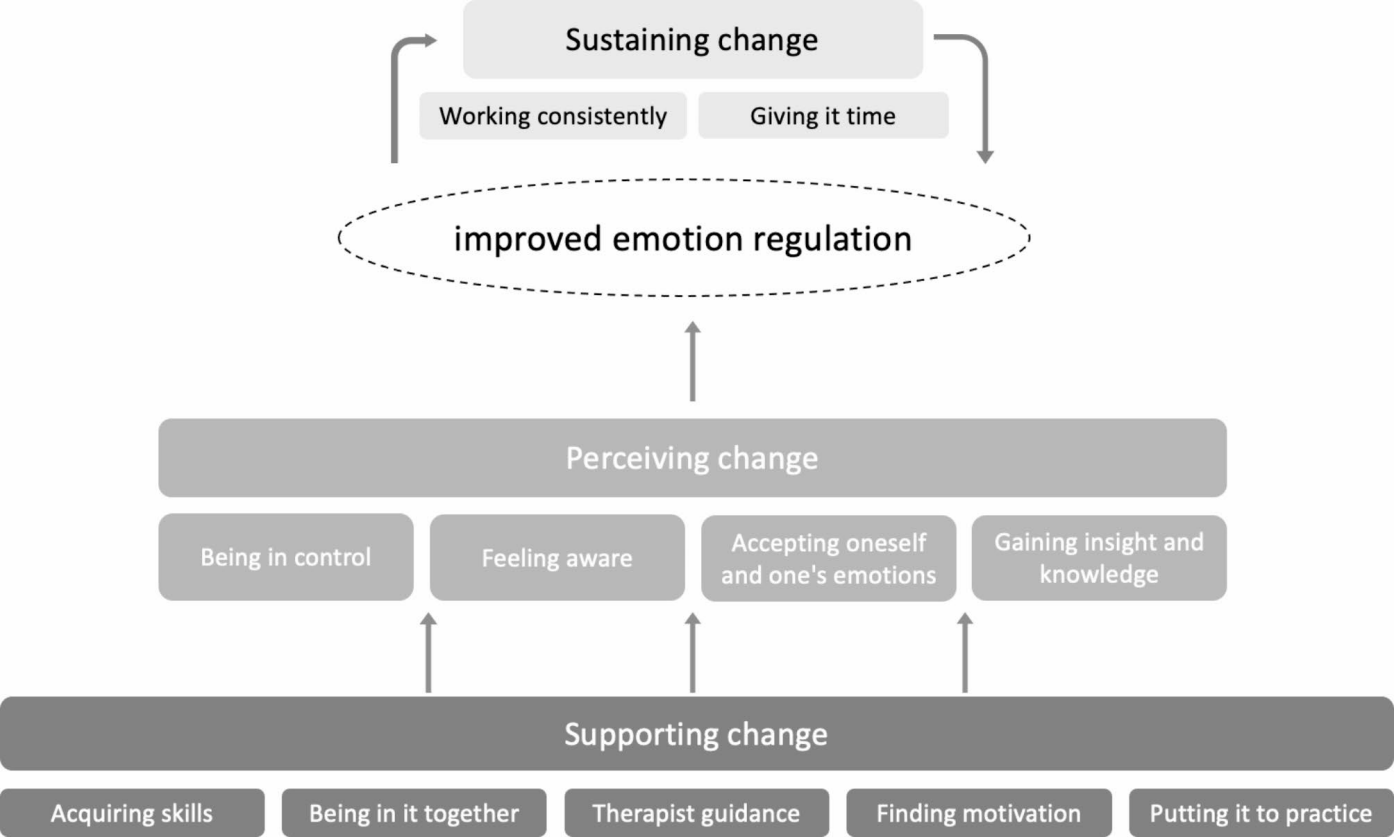
Model of change including core themes and subthemes

The thematic analysis resulted in three core themes capturing the participants’ experiences of change (see Table 2 for an overview). The first core theme, ‘*perceiving change*’, represents the specific changes the participants experienced, with four subthemes: 1a) *being in control*,* 1b) feeling aware*, 1c) *accepting oneself and one’s emotions* and 1d) *gaining insight and knowledge*. The second core theme, ‘*supporting change*’, captures factors that supported these changes, with five subthemes: 2a) *acquiring skills*,* 2b) being in it together*,* 2c) therapist guidance*,* 2d) finding motivation,  and 2e) putting it into practice*. Finally, the third core theme, ‘*sustaining change*’, captures aspects important to maintain change, with two subthemes: 3a) *working consistently,* and *3b) giving it time*. See Fig. 1 for a model illustrating how the core themes and subthemes are interrelated.

**Table 2:** Core themes and subthemes

Core themes	Subthemes
1) Perceiving change	1a) Being in control 1b) Feeling aware 1c) Accepting oneself and one’s emotions 1d) Gaining insight and knowledge
2) Supporting change	2a) Acquiring skills 2b) Being in it together 2c) Therapist guidance 2d) Finding motivation 2e) Putting it into practice
3) Sustaining change	3a) Working consistently 3b) Giving it time

### Theme 1: perceiving change

The first theme represents the participants’ experiences of change. Most of the participants described having undergone some kind of change following the intervention. The degree of change varied among the participants, with some participants also stating that they were uncertain whether they had experienced any change or said they would need more time to know whether they had changed. The changes were classified into the subthemes “being in control”, “feeling aware”, “accepting oneself and one’s emotions”, and “gaining insight and knowledge”.

#### Subtheme 1a: being in control

Several participants expressed an improved ability to control their emotions following their participation in the intervention. More emotional control also appeared to be related to feelings of calmness or emotional stability. This change was something that the participants themselves noticed, but also something others had observed. One participant said the following:

*“I feel that I have more control over my emotions*,* or in a way*,* control over myself with emotions. My partner also says that she notices a change in that*,* I am much calmer*,* or that I am able to hold off a bit more*,* so she says it is starting to get better” (Participant 1).*

In line with having more emotional control, some participants also expressed an improved ability to delay emotional reactions if they were not appropriate in a certain situation. At the same time, it was emphasized that they still acknowledged their emotions, and as such, it was different to suppressing, or pushing down, one’s emotions. One participant described:

*“If I feel that it is not the right time for that reaction to come*,* then I can let it come and then just let it pass by*,* and then I can revisit it later*,* because*,* okay*,* it is not the right time now… But I must deal with them when the time is right because what I did before*,* was just to push them down and never bring them up again” (Participant 2).*

One participant described that they previously assumed that their emotional responses were more fixed traits, whereas now they had learned that their emotions were more manageable, as illustrated by the following quote:

*“I have noticed that I can*,* instead of jumping straight to a reaction*,* I can take some time to think about my reaction*,* and that is something I have done very little of in the past. Because I have been like: ‘I am probably just someone who reacts’*,* but then I have realized that you can change those patterns of behavior. Even if you have a certain personality type*,* you do not always have to act on the emotions that arise” (Participant 3).*

Taken together, this subtheme illustrates how participants experienced changes that related to emotional control, including being able to inhibit emotional responses and experiencing their emotions as more manageable.

#### Subtheme 1b: feeling aware

In the interviews, several participants described an improved awareness of their emotions. It was further described that they were more present and able to be in the “here-and-now”. This kind of awareness was stated to be helpful when they were stressed or overwhelmed, by bringing their attention back to their emotions or their bodily sensations. They described that they would more frequently take pauses throughout the day to “check in” with themselves. One participant said:

*“To my relief*,* I notice several times a day that I kind of*,* where are you now*,* what are you feeling now*,* what is happening now?” (Participant 4).*

Several participants emphasized that they were more aware of their emotions, and they were better at identifying, recognizing, and labelling their emotions. One participant expressed:

*“I am better at naming emotions*,* yes*,* or just being aware that they are there. That was important*,* that was something I benefitted from” (Participant 4).*

Consistent with this, one participant described how they had become more aware what emotions they were feeling, as well as the difference between primary and secondary emotions, as illustrated by the following quote:

*“Often a lot of emotions would be expressed as anger*,* especially towards the people that are close*,* because that was an emotion that I had more control over*,* but it was perhaps fear or sadness I was feeling*,* so I have become a bit more aware of that” (Participant 3).*

In sum, this subtheme shows how participants experienced a higher awareness of their emotions, where they described an improved ability to notice and identify their emotional states.

#### Subtheme 1c: accepting oneself and one’s emotions

Acceptance seemed to be central in the participants’ accounts, including both self-acceptance and emotional acceptance. With this acceptance, the participants also seemed to have a higher tolerance or an improved ability to endure their emotions. One participant said:

“*To sort of say*,* emotions are not dangerous*,* like*,* the emotion in itself is not dangerous*,* they are just there*,* right?” (Participant 2).*

Rather than attempting to suppress or battle their emotions, the participants reported adopting a more accepting approach, where they allowed themselves to simply observe and acknowledge their emotions without judgment. One participant described:

*“If I am in a negative position*,* then I kind of remind myself: “okay*,* why am I doing this*,* why do I feel this?” (Participant 1).*

Some participants described an increased self-acceptance, accepting both positive and negative sides of themselves. They reported to be less self-judgmental and more forgiving of themselves or mistakes they had made. Accepting oneself also seemed to help with coping. One participant said:

*“It seems almost paradoxical to accept something you wish you didn’t have. But I handle it better by accepting how it is” (Participant 4).*

Taken together, this subtheme highlights how participants experienced an increased acceptance of themselves. The participants adopted a more accepting approach to their emotions, where they would observe and acknowledge their emotions, with less judgement.

#### Subtheme 1d: gaining insight and knowledge

Several participants reported to have gained insight, including insight about oneself, such as one’s own challenges and coping strategies, but also knowledge about ADHD, emotions, and emotion regulation strategies. One participant said:

“I have gotten more insight into how to deal with these things” (Participant 5).

The participants came into the intervention with varying degrees of knowledge and previous experiences. Some participants had been diagnosed with ADHD for a long time, and had previous experience with therapy, whereas others had just received the diagnosis. As such, some of the participants who had been diagnosed for a longer time expressed that they already had knowledge of the course material. In line with this, one participant described the following when asked about experienced changes:

*“I use these kind of tools all the time. I did that before as well*,* but maybe I did not know that it was that I was doing*,* so it has been nice to become aware of what kind of tools I am actually using” (Participant 6).*

One participant expressed how gaining insight into ADHD-related challenges helped her to understand that these challenges were not due to personal failings:

*“It is not me as a person that is wrong or should have been different or something like that*,* but these are actual real problems that those with ADHD generally struggle more with than others” (Participant 7).*

In sum, the participants reported to have gained more insight and knowledge following their participation, which appeared to be related to an improved understanding of themselves and their challenges.

### Theme 2: supporting change

The second core theme centers around the elements that facilitated the changes the participants experienced. While the participants identified various factors that supported their process of change, it was also apparent that these same elements could hinder their progress if not applied effectively, or the same factors could be perceived as helpful by one participant, but unhelpful by another. The current theme was classified into five subthemes: “acquiring skills”, “being in it together”, “therapist guidance”, “l finding motivation” and “putting it into practice”.

#### Subtheme 2a: acquiring skills

Most participants highlighted the intervention’s specific content, such as the skills and psychoeducation, as key facilitators for change. Many emphasized the value of the stop / check-in skill, which encourages the participants to take regular pauses to check in with themselves, considering their emotions and thoughts, and making more mindful decisions. At the same time, there was notable variation in what specific tools or skills the participants found to be beneficial. Due to the heterogeneous nature of ADHD, the participants pointed to the importance of having a wide selection of skills. One participant said:

“*We got some tools each time, and those tools, getting a lot of those emotion regulation tools, that has been very nice, because due to that, I was actually able to find a mindfulness skill that worked for me” (Participant 1).*

Several participants stated they had been skeptical of parts of the intervention beforehand, especially the parts about mindfulness. In this regard, it was deemed as beneficial that the therapists underscored that it takes time to succeed and to not have unrealistic expectations. One participant said:

“*The first time I tried it*,* I was like: “it probably won’t change much*,* but let’s give it a try”. But I actually felt quite an immediate response. So*,* there were several things that you could try that had better effects than I expected before trying them” (Participant 3).*

Some participants also emphasized the importance of other content, such as psychoeducation or theories on emotions. For some, this contributed to new insight which again could lead to improved management of emotions, as described by the following quote:

*“We talked a lot about*,* you know*,* what kind of person you want to be and the goals you want to set*,* and the choices you make and the actions you take being useful in reaching that goal. That has helped. For example*,* when I have had discussions with my partner or there have been situations where emotions tend to*,* you know*,* become difficult to control*,* then I have thought: ‘well it is not helpful if I say all of that now’*,* and that I need to think about it bit more*,* you know” (*Participant 3*).*

At the same time, the skills and psychoeducation could also be experienced as a barrier by not being tailored to ADHD, or if one felt the skills were not relevant to oneself. For instance, one participant mentioned that they did not find the skills fitting for people with ADHD. This participant underscored that it is important that the content of the intervention is both specific and feasible for adults with ADHD to be helpful:

*“I wonder if it perhaps was the skills that I did not think were tailored to us… because just having to*,* what was it*,* one of them was: “notice when you brush your teeth and feel every tooth” uh*,* and I do not have time for that’” (Participant 6).*

To summarize, most of the participants found specific content or skills presented in the intervention as valuable. However, some also felt that the intervention content was not entirely relevant or tailored to ADHD.

#### Theme 2b: being in it together

The value of being together with other people with ADHD in a group seemed to be central in the participants’ experiences. Some participants highlighted this as the most important aspect of the intervention. The group’s real-life examples of living with ADHD seemed to color the content of the intervention and strengthen its connection to ADHD. Most of the participants reported to value the group discussions and being able to talk about their own experiences in a group with others who understood their challenges. One participant said the following:

*“Something that made a huge difference was simply meeting like-minded people in a group*,* and the dialogue that unfolded from that*,* I would almost dare to say*,* that has been a gamebreaker for me” (Participant 8).*

Meeting others with ADHD in this setting was reported to contribute to an experience of universality, which made the participants feel validated and less alone in their struggles. One participant said:

*“You meet others in the same situation*,* and there can be this kind of belonging with such a group… That has a lot of value” (Participant 9).*

Being in a group was reported to make the intervention more relatable, where the examples from other group members helped make the program more relatable to real life. Through the other participants, they would get examples of how the skills could be applied in everyday life. Some also expressed that being with others made it easier to learn. One participant said the following:

*“I use some apps and such*,* but it still is not the same. If you can talk to others and they share experiences and tips and so on*,* it is a bit different. I think you learn much more from that than always trying to figure things out on your own” (Participant 9).*

At the same time, the group could also present challenges. For example, some participants said it could be difficult to relate to the other group members at times, or it could be a challenge to be vulnerable in such a setting with new people. One participant also noted that the group could exaggerate their ADHD symptoms.

*“When you’re around others who have ADHD*,* you talk about it*,* and you kind of*,* it’s like you sort of like some of the symptoms get intensified. It passes*,* in a way*,* but suddenly I found myself forgetting things and becoming distracted” (Participant 3).*

Many participants expressed that there should have been more time for group interaction and peer discussions in the program, including discussions about the course content, but also about personal life experiences.

In summary, the other group members were highly important to most participants. The participants valued discussions with peers who understood their perspectives and shared similar experiences, making them feel less alone.

#### Subtheme 2c: therapist guidance

The third subtheme focuses on the role of the therapists. The therapists could both facilitate and hinder change, where a therapeutic match seemed to be crucial. It was emphasized that it was important to have therapists ‘who know what they are doing’. The therapists could be thought of as navigators, guiding the participants through the intervention, and ensuring they were on the right path. One participant said:

*“The ones leading it*,* those who guided the conversation*,* they managed to keep it grounded. The insights they shared were valuable. There was a progression in it” (Participant 8).*

Some participants expressed that it was helpful to have therapists that they could go to for advice and talk to if they needed help. The therapists seemed to offer a different perspective on things, which was valuable. One participant said:

*“They were really good at it*,* those who facilitated it. They could*,* in a way*,* or at least I found it very helpful*,* that they always had a different*,* how can I say this*,* when I expected a yes or no answer*,* they always had another perspective in a way” (Participant 9).*

At the same time, there could also be challenging aspects related to the therapists. For instance, one participant expressed that the therapists did not have enough understanding or compassion for their challenges, which made it difficult to be open:

*“I felt a bit*,* in a way*,* not taken seriously when we tried to give examples of how intense it could be. We all read signals*,* right? So*,* once you get that feeling*,* well*,* yeah*,* nothing more comes out*,* yeah*,* we don’t open up much more”* (Participant 6).

Some participants expressed that the therapists did not have lived experience with ADHD, which could make it difficult for the therapists to truly relate to the participants’ experiences. One participant said:

*“If you are trying to understand someone else’s experience of something*,* it can be quite challenging at times… Like*,* you must have felt it in your body. That is how it is with all things; you cannot imagine what it is like to have a broken leg before you have broken it yourself”* (Participant 8).

In accordance with this, it was suggested to involve someone with ADHD as group leader to provide personal examples on how to utilize the skills presented in the program.

In sum, the therapists were seen as navigators, offering insight and guidance throughout the course of the program. Having a therapist that showed compassion and understanding was important, where some participants emphasized that therapists without ADHD might struggle to understand the participants’ experiences.

#### Subtheme 2d: finding motivation

The fourth subtheme highlights how internal processes, such as motivation and willingness to engage in the process, were essential for positive outcomes. These were also aspects that could potentially hinder the therapeutic process. Several participants emphasized that it was difficult to maintain motivation throughout the program. One participant said:

*“I lacked a bit of willpower towards the end” (Participant 1).*

Some participants further expressed how taking part in psychological interventions, and especially in an intervention with an emphasis on emotions, could be demanding. As such, one had to be willing to engage, as participating in the intervention also had a cost. One participant said:

*“It costs something to get into it. It costs to sit down and take a dive into something that might not be so comfortable*” (Participant 7).

At the same time, it was underscored that perhaps it was these “uncomfortable” aspects that contributed to progress:

“*There were things that I would not have thought about doing myself*,* that I thought*,* there and then was a bit “oh*,* that is uncomfortable” or “I cannot be bothered to” or “that sounds difficult” or something like that*,* but then it was actually quite useful*,* and it is perhaps those things I thought about most afterwards*,* that made me think in a slightly different way” (Participant 9).*

Taken together, motivation was seen as crucial to achieve therapeutic change, but also as challenging to maintain throughout the intervention.

#### Subtheme 2e: putting it into practice

The last subtheme highlights that change requires practice of skills outside the therapy setting and in everyday life situations. Although this was deemed as highly important to make a change, most participants described the practice and implementation of skills into everyday life as difficult.

To be implemented in everyday life, it was important that the skills were perceived as relevant and useful. The participants expressed a great heterogeneity in their preferred skills, and some expressed that they had to adjust the skills to make them ‘work for them’. Moreover, a wide selection of skills that could be practiced ‘on the go’ were experienced as useful by the participants. One participant said the following:

*“We had a lot of different skills and then there were things you could do in different ways. It was not like you had to sit down and breathe*,* you could practice when you were walking*,* driving or all kinds of things*,* and that makes it more accessible*,* then you do not have to set aside time*,* you can just do it wherever you are*,* so that has been nice” (Participant 3).*

At the same time, it could be challenging to implement skills training in everyday life. Some participants pointed to ADHD-related challenges, such as problems getting started, inattention, or having a ‘wandering mind’, as reasons for not doing the skills training. The lack of skills training could also be related to the program itself, for instance, some participants pointed to unclear instructions of the skills or not finding the skills suitable for their needs. Other reasons included external events, such as having to prioritize work or family life. One participant said the following when asked about challenging aspects of the program:

*“To do the tasks*,* or in a way keeping the program alive when you are not there. Because life gets you*,* with kids and activities and everything there is*,* and in a way setting aside time for it” (Participant 7).*

The companion app, which was intended to help the participants with skills training in between the group sessions, could be experienced as both a facilitator and a barrier. With regard to helpful aspects, several participants found the combination of face-to-face and digital elements as useful, where each of the elements had its own value. In particular, the participants mentioned that it was helpful to have access to the skills via the app and to receive reminders on their phones. One participant said:

“*We always received a message*,* which was good… it really helped me to remember because otherwise*,* I would have typically postponed it or forgotten*,* but now*,* I did it quite often after receiving the message*” (Participant 9).

There were also challenging aspects related to the companion app. For instance, several participants struggled with the login procedure, as it required two-factor identification. This was highlighted as a barrier, where the login procedure to the app was deemed too complicated and time consuming. One participant said the following:

“*I think one of the main reasons I used it (the companion app) so little was because it became burdensome*,* especially for a group like us*,* who are always short on time and tend to give up when faced with resistance” (Participant 6).*

In sum, using the skills from the intervention in their everyday life was important for change. To facilitate this kind of implementation, it was essential that the skills were relevant and accessible.

### Theme 3: sustaining change

The third core theme focuses on making the change last. It was emphasized that change was a continuous process which was not complete after the program had ended. This theme has two subthemes: “working consistently"  and “giving it time”.

#### Subtheme 3a: working consistently

Several participants emphasized that to achieve and maintain change, it required consistent work, where repetition and continuous practice of the skills were necessary. It was also expressed that more work was required to obtain a lasting change. One participant said:

*“A lot more work is needed*,* but at least I notice that it is going in the right direction” (Participant 2).*

It was highlighted that maintaining change required a change in habits, where the skills needed to become integrated into their everyday life and routines. One participant said:

*“You are essentially taking part in a lifestyle change to improve your well-being. So*,* you have to work on things more or less continuously”* (Participant 5).

There were also some participants who expressed that they had not experienced any changes yet, and they would not know whether the program was effective until they had put down more work, as illustrated by the following quote:

*“But if I notice a change*,* I mean*,* I believe that if it is going to have an effect*,* you have to continue with it” (Participant 9).*

Taken together, participants highlighted that change required consistent work, where some also felt that more work was needed in order to benefit from the intervention.

#### Subtheme 3b: giving it time

Several participants expressed that these kinds of life changes take time and that they needed to continue with the program over time. Some participants felt that the length of the program was too short to achieve change within the timeframe. One participant said:

*“I feel that eight weeks is a bit too short to establish new habits” (Participant 2).*

To help maintain the effects of the program, one participant suggested that there could have been a type of “aftercare” following the end of the program or to allow the participants to retake the program after some time. One participant expressed:

*“We could have had a group like that running for a long time*,* that it was continuous. I don’t know*,* I think it was*,* maybe not every week*,* but it is probably very useful for many to have something like that*,* a coach or*,* at least something similar” (Participant 9).*

In line with this, it was also suggested to increase the number of group sessions or add more time to the sessions so that the participants had more time to repeat and discuss the content. One participant said:

*“I wish there was more time*,* more time to go through things*,* more time for reflections around the tasks you were given or were about to get started on” (Participant 8).*

In sum, this subtheme illustrates that change takes time. With regards to this, some felt more long-term support would have been beneficial to maintain change.

## Discussion

This study explored the first-person perspective of change among adults with ADHD who had participated in a blended intervention targeting emotion dysregulation. More specifically, the study aimed to understand the participants’ experiences of change and identify contributors to change. The thematic analysis resulted in three core themes, (1) ‘perceiving change’, (2) ‘supporting change’ and (3) ‘sustaining change’. Taken together, the findings highlight how the participants experienced changes in how they related to and managed their emotions. These changes were supported by a combination of internal factors such as motivation and engagement, and external factors, including the skills of the intervention, other group members and the therapists. The findings further shed light upon the temporal aspect of change, where the participants experienced change as a gradual process.

The participants described changes within the domains of control, awareness, acceptance, insight, and knowledge with regards to themselves and their emotions following their participation in the intervention. These findings are in line with findings from a qualitative study on a DBT-based intervention for adolescents with ADHD, where the participants described increased knowledge, awareness and acceptance of themselves [45]. Moreover, Janssen et al. [35] also found aspects such as “insight” to be a part of the process of change among adults with ADHD in a mindfulness-based intervention. While these aspects were described as the experienced outcomes by the participants in our study, they might also be understood as components underlying a more general change in emotion regulation, where these domains can be viewed as facets of emotion regulation. For example, Gratz and Roemer’s [46] conceptualization of emotion regulation includes “the awareness and understanding of emotions”, “the acceptance of emotions”, “the ability to control impulsive behaviors”, as well as “the ability to use situationally appropriate emotional regulation strategies” (p. 42). Gratz and Roemer [46] further underscore that impairment of any of these abilities leads to emotion dysregulation. As such, the experienced change from the participants fits within existing conceptual frameworks of emotion regulation [46] .

The findings highlight several factors that supported the participants in their process of change. Although these are personal experiences that cannot be generalized as facilitators for change, these experiences point to aspects of the intervention the participants themselves experienced as important in the change process [47]. Firstly, acquiring coping skills was deemed as important, but at the same time, there was a large variety in the specific skills that each participant found useful. As such, having a wide selection of coping skills in the intervention was beneficial. This is line with findings from Janssen et al. [35] where the participants also highlighted diversity of exercises as a facilitator in a mindfulness-based intervention for adults with ADHD. Moreover, it was emphasized that the skills should be easy to implement in everyday life and tailored to the target group. With regards to this, skills that could be practiced ‘on the go’ were deemed as beneficial. Thus, these findings offer some recommendations on how skills or other therapeutic content can be presented and implemented in psychological interventions for adults with ADHD.

The support from others, including other group members and therapists, seemed to be of importance across the participants’ reports. In particular, peer support was highlighted as valuable, which aligns with findings from previous studies on psychological interventions for ADHD [35, 36, 45, 48, 49]. Meeting others with ADHD made the participants feel less alone in their struggles and validated their experiences. This finding is related to the concept of ‘universality’, which is considered a key therapeutic factor within group therapy [50]. The participants further expressed that support from the therapists was of significance. This aligns with the general literature on psychotherapy, which has pointed to factors such as therapeutic alliance as being critical to therapeutic change [51]. Interestingly, some participants emphasized that the therapists did not have lived experience with ADHD, which could make it difficult for the therapists to relate to their experiences. As such, one suggestion was to include someone with ADHD as co-facilitator in the groups. Within mental health interventions, some studies have reported peer-based co-facilitation interventions to have beneficial effects on engagement and clinical outcomes [52, 53]. Seery et al. [36] also found that the inclusion of a facilitator with ADHD in an online intervention for adults with ADHD was experienced as beneficial by the participants, for instance by making the program more relatable and credible. As such, the findings suggest that incorporation of peers in psychological interventions for adults with ADHD can be perceived as beneficial.

The participants further pointed to more intrinsic factors as contributors to change, such as their motivation and willingness to engage in the intervention. This aligns with previous research, showing that motivation and engagement are central for change within psychotherapy [54, 55]. At the same time, motivation appeared to be challenging for most participants, particularly regarding homework assignments. Adherence to homework is a general challenge in psychological interventions, with many participants struggling to adhere to homework assignments [56, 57]. Homework has also been reported to be a specific barrier in psychological interventions for adults with ADHD in previous studies [35, 37]. While some participants in this study described factors such as motivation or other intrinsic factors as barriers to homework completion, others pointed to issues related to the intervention itself, such as the tasks not being tailored enough to ADHD, or a burdensome login procedure to the companion app. As such, these findings point to the importance of making adaptations to the intervention to facilitate homework completion and make the intervention more ADHD-friendly. Adaptions could include simplifying the login procedure of the companion app with the use of biometrics and by making the companion app more adapted to the person’s preferences and routines.

The findings showed that in order to maintain progress, the participants experienced that consistent and continuous work was required. In line with this, the participants asked for long-term support or aftercare following the end of the intervention. A preference for more long-term support aligns with previous findings and fits with the persistent nature of ADHD [2, 35]. At the same time, long-term support can be challenging to provide, as it requires additional clinical and economical resources. One possible solution could be to use the companion app to provide continuous support, where the app might also facilitate communication between the group members, enhancing its utility and support capabilities.

### Implications

This study provides insight into experiences of changes among adults with ADHD who had taken part in a blended intervention aimed at improving emotion dysregulation. The study further highlights perceived contributors to change. For example, the participants emphasized the group-based approach, a wide selection of coping skills, and skills that could be practiced ‘on the go’ as useful aspects of the intervention. Moreover, the findings also give some suggestions for improvement that could make the intervention more ADHD-friendly, with some examples of specific adaptations, such as: (1) tailoring the intervention more to ADHD, for instance by including skills and psychoeducation that are more specific to ADHD; (2) inclusion of a co-therapist with lived experience with ADHD, which may offer a unique understanding of the participants’ perspectives; (3) simplifying the login procedure to the companion app by implementing biometric verification, thereby improving its accessibility; (4) making the companion app more personalized, for instance by letting participants choose timing of reminders, (5) allowing for communication between group members within the companion app to provide additional support, and (6) inclusion of more long-term support beyond the intervention period to maintain progress, for instance by adding “aftercare” modules in the companion app or organizing follow-up group sessions.

The findings also highlight that it might be useful to include more ‘growth-based’ outcome measures in intervention studies for ADHD. For instance, many participants described changes in relation to coping abilities, such as improved skills, knowledge, or acceptance of one’s challenges. Such aspects are often overlooked in intervention studies for adults with ADHD, which predominantly focus on reduction in core psychopathology. Therefore, it might be beneficial for future studies to include outcome measures assessing aspects like coping abilities, as this may reflect an area of change among the participants.

### Trustworthiness and limitations

The authors have strived to give a transparent and thorough overview of the research process, including data collection, data analysis, and interpretation, to ensure credibility and dependability of the findings. To improve transferability of the findings, a detailed description of the procedure is provided in the final report. We have further included several illustrative quotes from the participants across all subthemes in the final report, so that the readers themselves can evaluate our interpretations of the data material. Importantly, the researchers made sure to engage in a continuous reflexive process of evaluating their own interpretations, perceptions and pre-knowledge, both individually and together during the analytic meetings. The involvement of several researchers in both the coding and analysis of the data further contributed to the inclusion of diverse analytic perspectives. For example, during the initial analytic meeting between the first and last author, they discussed how their varying backgrounds led to them having different entries into data material. The last author, who approached the data with a non-clinical perspective, was able to emphasize aspects of the data that differed from those identified by the first and second authors. This perspective served to complement the more clinically oriented viewpoints, enriching the analysis and improving the trustworthiness of the findings.

The study also has some limitations that should be noted. First, only participants who completed the intervention were included. Thus, we lack the perspective of the participants who dropped out. This information could be important in order to gain a deeper insight into possible barriers for intervention completion and adverse effects. The relatively small sample size, consisting of nine participants, could also be considered a limitation. For the current study, the pool of available participants was restricted, as the participants had to have taken part in the feasibility study of ERIA. The small sample size, together with interviews that were somewhat brief in nature, may raise some concern regarding saturation. Although nine participants are generally seen sufficient to reach code saturation (i.e., identification of the relevant thematic issues), some argue that a sample size of minimum 16 participants is needed to reach meaning saturation (i.e., when the researchers truly understand these thematic issues) [58]. At the same time, Braun and Clarke argue that it is difficult to operationalize data saturation or to give guidelines on how many interviews are sufficient in a thematic analysis [59]. Despite limitations in sample size, we were able to identify various examples, variations and nuances within and across the themes, which we believe support a richness in the data material. The current sample also had high sample specificity, as all participants had all undergone the same intervention, making the current sample more homogeneous, and thus increasing the information power [60].

Another possible limitation is the potential bias among the authors, where three of the authors were involved in the development of the intervention. This may have led the authors to be biased in their interpretations, overestimating the positive effects of the intervention. At the same time, in-depth knowledge of the intervention among the researchers could be considered a strength, as the researchers’ familiarity with the intervention could have facilitated a deeper understanding and provided more context to the participants’ narratives. Moreover, potential bias does not need to be a limitation within qualitative research if the authors uphold reflexivity. The authors have carefully considered and reflected upon their own preconceptions and interpretations in the research process. Moreover, the current investigation also involved co-researchers who were not involved in the intervention development, which could help to improve overall credibility.

## Conclusions

This study provides insights into the first-person perspective on experiences of change and contributors to change in a blended intervention for emotion dysregulation. The findings show that the participants experienced various changes with regards to how they managed their emotions. These changes appeared to be supported by a combination of internal factors such as motivation and willingness to engage with the intervention, and external factors, including the intervention content, other group members and the therapists. Moreover, the participants emphasized that change was a gradual process that requires consistent work. The findings of the study should be valuable to the development of future psychological interventions for adults with ADHD.

## Data Availability

The datasets generated and analyzed during the current study are not publicly available due to the sensitive nature of the data but are available from the corresponding author on reasonable request.

## Abbreviations

ACT: Acceptance and commitment therapy
ADHD: Attention-deficit hyperactivity disorder
DBT: Dialectical behavior therapy
DERS: Difficulties in emotion dysregulation scale
ERIA: Emotion Regulation Intervention for ADHD
TAU: Treatment-as-usual
